# Interpreting weaning success and ventilation duration in PRiVENT

**DOI:** 10.1186/s13054-026-06420-z

**Published:** 2026-10-26

**Authors:** Huili Cheng

**Affiliations:** Department of Ophthalmology, Wuhan Asia General Hospital, Wuhan University of Science and Technology, No. 300 Taizihu North Road, Wuhan Economic and Technological Development Zone, Wuhan, Hubei 430056 China

Successful liberation from invasive ventilation and a shorter treatment course are related but distinct goals. Trudzinski and colleagues’ PRiVENT study provides a useful setting in which to consider both [1]. The specialist-led programme was feasible across a regional intensive care network. Its clinical interpretation requires considering the weaning association alongside outcome classification, treatment duration, and resource use.

The intervention analysis set comprised 349 patients, with 1,768 controls. Successful weaning was observed in 201/345 intervention patients (58.3%), with four outcomes missing, versus 914/1,768 controls (51.7%). The adjusted primary odds ratio was 1.46 (95% confidence interval, 0.99–2.17; *P* = 0.057). This interval includes no difference as well as clinically important benefit; it does not establish ineffectiveness. The prespecified matched analysis was more favourable, with an adjusted odds ratio of 2.90 (95% confidence interval, 1.61–5.24). Both analyses arose from the same nonrandomized study and remained subject to residual confounding. The stronger matched association supports the possibility of benefit without establishing a causal treatment effect.

Outcome comparability involves both ascertainment accuracy and the clinical event being classified. Intervention outcomes were prospectively documented, whereas control outcomes were reconstructed from insurance claims. The primary outcome was liberation before discharge from the intensive care unit or an affiliated weaning centre. Stays exceeding six months after ventilation began were classified as failure. The main Methods classify death before successful weaning as failure. Supplementary Appendix S2 to the PRiVENT report [1] lists death before discharge from the index hospitalization as failure. Could the authors clarify whether death after successful weaning but before discharge from the index hospitalization was classified identically in both cohorts? Clarification of how intensive care unit, weaning-centre, and index-hospital discharge were aligned would also help. These wording differences do not establish actual misclassification or its direction, but clarification would make the clinical meaning of the comparison more explicit.

The authors also report a competing-risk analysis of time to successful weaning. After adjustment, the subdistribution hazard ratio was 1.12 (95% confidence interval, 0.91–1.38; *P* = 0.30). This complementary analysis did not establish a clear difference in the cumulative incidence of weaning when accounting for death. It should be interpreted separately from ventilation duration, which addresses another aspect of the treatment course.

Using observed ventilation hours within 30 days, median duration was 456 h in the intervention cohort versus 342 h in controls. The model-based adjusted difference was 81 h (95% confidence interval, 49–114). Longer duration was also reported under the alternative definition assigning ventilation time through day 30 to patients who died earlier. Transfers to specialist weaning centres occurred in 57/349 intervention patients (16.3%) and none of the controls. This pathway difference provides context, but does not show that transfer caused longer ventilation. Longer observed ventilation indicates greater treatment exposure, not necessarily greater patient burden or harm. The in-hospital mortality comparison was also inconclusive (adjusted odds ratio, 0.93; 95% confidence interval, 0.64–1.35).

The programme required specialist boards, bedside consultations, education, and coordination with weaning centres. As the authors acknowledge, these inputs must be considered alongside any clinical gain. The planned economic evaluation will be important for determining whether potential benefit justifies the additional specialist input and treatment time. The current clinical results alone do not settle that balance.

PRiVENT supports the feasibility of regional specialist weaning support and suggests potential clinical benefit, consistent with the authors’ cautious conclusion. Keeping discharge-based weaning success, survival, ventilation duration, and resource use together would make its implications for programme adoption clearer. A cluster-randomized evaluation with consistent outcome ascertainment could address this balance without treating either a higher success proportion or longer ventilation as sufficient evidence of net benefit or harm.

## Data Availability

No datasets were generated or analysed during the current study.

## Abbreviations

PRiVENT: PRevention of invasive VENTilation

## References

[1] Trudzinski FC, Neetz B, Joveš B, Kempa A, Schneider A, Biehler E, et al. A specialist-led interprofessional weaning program to prevent weaning failure in high-risk patients: the PRiVENT study. Crit Care. 2026;30:507. 10.1186/s13054-026-06357-3. PMC13628980 42823746

